# Edible and Recyclable Gelatin‐Based Electronics for High‐Precision Health and Environmental Monitoring

**DOI:** 10.1002/advs.202507950

**Published:** 2025-08-04

**Authors:** Ming Dong, Pietro Cataldi, Han Zhang, Emiliano Bilotti, Conor Boland, Athanassia Athanassiou, Dimitrios G. Papageorgiou

**Affiliations:** ^1^ School of Engineering and Materials Science Queen Mary University of London London E1 4NS UK; ^2^ Smart Materials Istituto Italiano di Tecnologia Via Morego 30 Genova 16163 Italy; ^3^ WMG University of Warwick Coventry CV4 7AL UK; ^4^ Department of Aeronautics Imperial College London Exhibition Road London SW7 2AZ UK; ^5^ School of Mechanical and Manufacturing Engineering Dublin City University Glasnevin Dublin D09 W6F4 Ireland

**Keywords:** activated charcoal, edible electronics, green electronics, multimodal sensors, recyclability

## Abstract

Edible electronics represent a transformative class of sustainable technologies that combine functionality, safety, and environmental transience. Here, a multifunctional, edible, and recyclable sensor film is presented composed of gelatin and activated charcoal—two naturally abundant and food‐safe materials—engineered for high‐precision health and environmental monitoring. These free‐standing composite films exhibit a phase‐separated bilayer structure, enabling the integration of an insulating gelatin top layer with a conductive charcoal‐rich bottom layer. At an optimal filler loading of 10 wt%, the films achieve a tensile strength of 60 MPa and electrical conductivity of 0.04 S m^−1^, supporting multimodal sensing of strain (gauge factor 3.8, response time 120 ms, stable over 10 000 cycles), humidity (40–95% RH), and temperature (0–90 °C). Demonstrations include real‐time motion tracking, respiration monitoring, speech recognition, and contactless thermal sensing. Uniquely, the films degrade fully in soil within three weeks or can be recycled in water without loss of mechanical or electrical performance. This work advances the design of sustainable materials that combine performance and sustainability, offering a scalable pathway toward circular electronics for a zero‐waste future.

## Graphical abstract

An edible and recyclable composite film composed of gelatin and activated charcoal is presented for multifunctional health and environmental monitoring. The edible films exhibit tuneable mechanical and electrical properties, along with multimodal sensing capabilities for strain, humidity, and temperature. The composite film is degradable and recyclable, supporting sustainable material development towards a zero‐waste future.

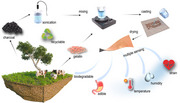

## 1 Introduction

The global market for electronic devices is rapidly expanding due to the increasing need for electronics in the areas of health monitoring,^[^ ^1^ ^]^ stretchable conductors,^[^ ^2^ ^]^ human–machine interaction,^[^ ^3^ ^]^ and sensors.^[^ ^4^ ^]^ However, the majority of electronic devices are fabricated using non‐biobased and non‐biodegradable materials, which are often rare and costly. This can result in challenges with sourcing and the accumulation of electronic waste (*e*‐waste) when discarded properly^[^ ^5^ ^]^ or worse, the proliferation of long‐lasting waste in the environment when disposed of improperly. Annually, 40 million tons of e‐waste is dumped in landfills worldwide, with projections indicating a surge to 120 million tons by 2050.^[^ ^6^ ^]^ Actions have been undertaken in materials, manufacturing, and recycling to reduce the use of hard‐to‐source and expensive elements, as well as to minimize the generation of *e*‐waste.^[^ ^7^ ^]^ One promising strategy to make electronics more sustainable and minimize *e*‐waste pollution is the development of green electronics^[^ ^8^ ^]^ produced with renewable and biodegradable materials that can interact with nature without leaving harmful byproducts or leaving a permanent footprint.^[^ ^9^ ^]^

Beyond green electronics, the field of edible electronics seeks to utilize the electrical characteristics of food and food additives to provide technological advancements, especially for gastrointestinal (GI) health monitoring,^[^ ^10^ ^]^ and tracking the growth or condition of edible products, reducing concerns about post‐harvest consumption. Compared to the more investigated ingestible electronics which rely on conventional electronic components,^[^ ^11^ ^]^ edible electronics promise to offer unique advantages, including: 1) the absence of hard‐to‐source and potentially harmful compounds, 2) natural biodegradability and/or non‐ecotoxicity, and 3) safety for ingestion without the need for medical supervision due to the lack of retention risk.^[^ ^12^ ^]^ This innovative approach not only promotes sustainable electronic usage but also reduces environmental impact by eliminating long‐lasting petroleum‐based materials and waste. Considering the intrinsic properties of materials used in edible electronics from a different perspective, it becomes evident that many of them are also suitable for applications such as environmental monitoring sensors. These sensors perform their function and then degrade in the environment, forming byproducts that are harmless to their surroundings.^[^ ^13^ ^]^ Such sensors would be of critical importance in agriculture and can enable the monitoring of critical endangered areas.^[^ ^14^ ^]^ Edible electronic devices are specifically designed to naturally decompose within the body or the environment after completing their intended functions, such as tracking, monitoring, and sensing. This unique characteristic makes them strong candidates for transient environmental monitoring applications, where devices can perform their role and then safely degrade without leaving harmful residues.^[^ ^15^ ^]^

The basic building blocks of electronic devices include insulators and dielectrics, conductors, and semiconductors. Insulators and dielectrics suitable for both edible electronics and environmental monitoring are abundant and include materials such as chitosan, albumin, gelatin, cellulose and its derivatives (e.g., ethyl cellulose),^[^ ^16^ ^]^ shellac resins, natural waxes, and more.^[^ ^17^ ^]^ However, conductors and semiconductors for these applications are much rarer, particularly when they must meet the requirements of both edible electronics and transient environmental monitoring simultaneously. Therefore, intensified efforts are needed to develop a comprehensive library of materials that can support these interdisciplinary tasks and drive technological advancements in this field.

Researchers have explored various edible electronic components, particularly edible conductors,^[^ ^17^, ^18^ ^]^ some of which are promising for both edible electronics and environmental monitoring. Edible conductors function through ionic conductivity, electronic conductivity, or a combination of both, depending on the underlying conduction mechanism.^[^ ^17^ ^]^ Ionic conduction, common in ion‐charged gels, offers significant potential for flexible strain sensors due to their excellent stretchability.^[^ ^19^ ^]^ For instance, gelatin‐based biogels have emerged as promising materials for soft robotics, offering flexibility, biocompatibility, and adaptability to complex movements.^[^ ^20^ ^]^ However, many ionic gels tend to dehydrate when exposed to air, becoming rigid, brittle, and non‐conductive over time, which compromises their stable sensing capabilities.^[^ ^21^ ^]^

On the other hand, electronic conductors achieve the required electrical conductivity for sensing applications by integrating conductive fillers into food‐grade matrices such as celluloses, proteins, and polysaccharides, offering the advantages of abundance, cost‐effectiveness, biodegradability, and edibility.^[^ ^22^ ^]^ Metal‐based, edible conductive fillers such as zinc, silver, and gold have been used for current collectors^[^ ^23^ ^]^ and electrodes in transistors.^[^ ^24^ ^]^ However, due to their high cost and persistence, these metals are often unsuitable for the large‐scale production of capillary sensors intended for dispersal in the environment, either before or after ingestion, making them less practical for applications such as environmental monitoring or agriculture. Additionally, intake safety restricts their daily consumption to microgram levels per kilogram of body weight.^[^ ^25^ ^]^

Alternatively, activated charcoal (AC) has gained recognition as an electrical conductor in edible electronics, offering benefits such as low cost, scalability for large‐scale production, adequate conductivity, and safe consumption at milligram levels per kilogram of body weight.^[^ ^25^ ^]^ As a result, various electronic conductors have been developed as composites using AC, finding applications in areas such as food monitoring,^[^ ^25^ ^]^ supercapacitors,^[^ ^26^ ^]^ electrochemical sensors,^[^ ^27^ ^]^ and triboelectric nanogenerators.^[^ ^16^ ^]^ However, current AC‐based edible conductors face challenges in mechanical strength, which limits their performance in specific applications.

Simultaneously, AC has demonstrated effectiveness in large‐scale environmental applications, such as water remediation and soil amendment, due to its exceptional capacity to absorb contaminants.^[^ ^28^ ^]^ While discussions continue regarding AC's potential secondary environmental impacts following extensive use in remediation,^[^ ^29^ ^]^ its application in electronic devices and sensors involves only minimal quantities—typically measured in milligrams per device, compared to the kilograms required for remediation or soil amendment. This dual functionality underscores AC as a compelling candidate for both edible electronics and environmental monitoring, effectively bridging the gap between these two fields and addressing the challenges of sustainability and scalability in both domains.

In recent years, numerous studies have focused on developing multimodal sensors capable of detecting stimuli such as strain, humidity, and temperature.^[^ ^30^ ^]^ These advanced sensors play an essential role in daily life by monitoring environmental and physiological conditions, contributing to enhanced health tracking and environmental awareness.^[^ ^31^ ^]^ However, most reported multimodal sensors are made from non‐biodegradable, inedible materials, posing potential environmental and health risks upon disposal.^[^ ^32^ ^]^ Importantly, although a variety of edible sensors have been developed, most are limited to detecting a single type of stimulus, such as strain,^[^ ^12a^ ^]^ tilt,^[^ ^33^ ^]^ pH^[^ ^34^ ^]^ and humidity.^[^ ^35^ ^]^ For example, in the work of Annese et al.,^[^ ^12a^ ^]^ an edible strain sensor was developed using a sprayable conductive ink composed of activated carbon and gummy candy, yielding a piezoresistive coating compatible with edible batteries and demonstrating gauge factors up to 92. Similarly, in the work of Rehman et al.,^[^ ^34^ ^]^ an edible and self‐powered humidity‐only sensor was fabricated using cellulose‐enriched rice paper, serving both as the sensing material and the tribopositive layer in a triboelectric nanogenerator, achieving a high sensitivity. Nevertheless, an ideal solution would be to develop an edible sensor that is safe for consumption, capable of detecting multiple stimuli, and environmentally harmless. Such technology would be a potential solution in a number of advanced applications.

This study presents a conductive composite film made from two safe, edible materials that decompose harmlessly in the environment. Electrically conductive AC particles were dispersed in water via sonication, combined with the natural biopolymer gelatin, and cast into a thin, free‐standing film. The resulting film features a phase separated, two‐layer structure, with a conductive AC layer at the bottom and an insulating gelatin layer on top. This dual‐phase composite film demonstrates robust mechanical properties and achieves the required electrical conductivity for multimodal sensing. It exhibits high sensitivity to strain, humidity, and temperature, enabling applications such as human motion and respiration monitoring, handwriting recognition, liquid nitrogen detection, and temperature and humidity sensing. Very importantly, the film is degradable in soil, leaving only minimal concentrations of inert and safe byproducts. It is also easily chemically recyclable using water without losing its electromechanical properties and is safe for ingestion at substantial intake levels.

**Figure 1:**
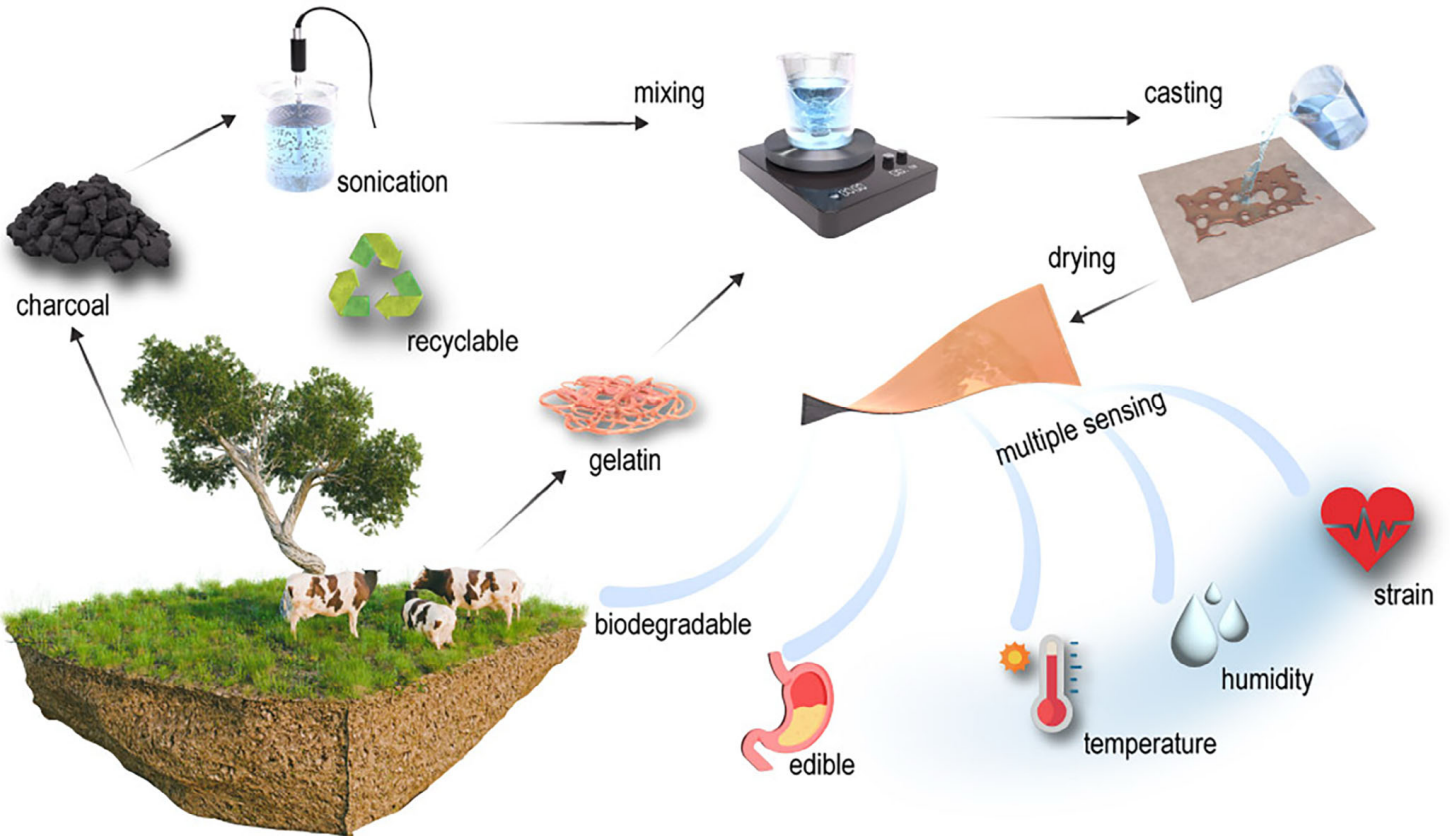
Preparation process for edible gelatin/charcoal composite films, illustrating steps including charcoal sonication in (deionized) DI water, blending with gelatin, and casting the composite films. The figure also highlights the films’ biodegradable, edible, and recyclable nature, as well as their multifunctional sensing capabilities.

In summary, the simple and sustainable water‐based fabrication process (**Figure** **1**), combined with the film's outstanding mechanical properties, exceptional capability to detect multiple stimuli, environmental degradability with harmless byproducts, recyclability, and edibility, represents a significant advancement in the fields of edible electronics and environmental monitoring. These free‐standing films pave the way for sustainable technologies, offering a viable, eco‐friendly alternative for future sensor applications.

## 2 Results and Discussion

### 2.1 Characterization of Charcoal and Gelatin/Charcoal Films

**Figure 2:**
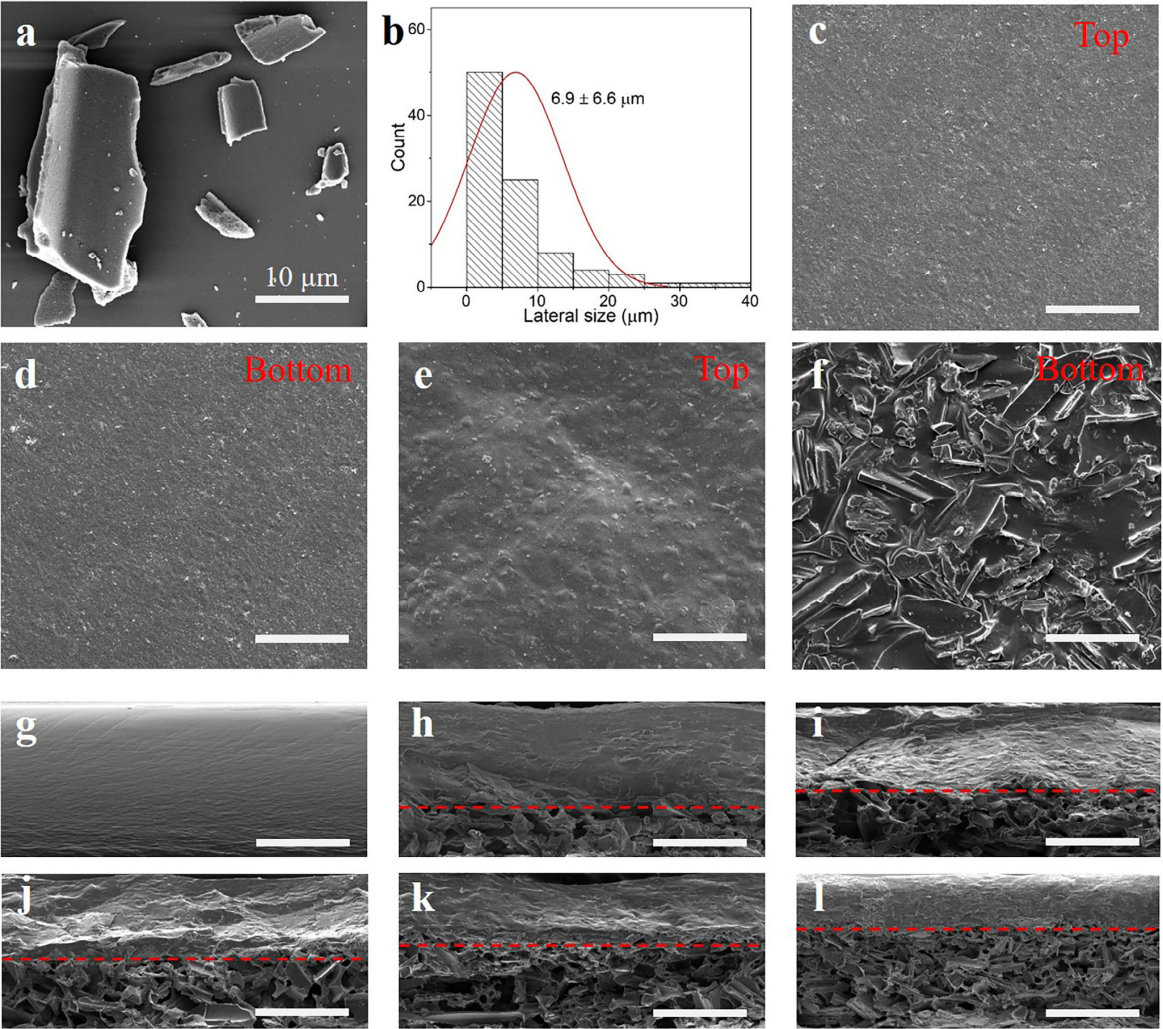
a) SEM image of charcoal particles. b) Particle size distribution of charcoal particles. c) Top and d) bottom surfaces of the pure gelatin film. e) Top and f) bottom surfaces of the gelatin/charcoal composite film containing 10 wt% filler. Scale bars represent 30 µm. Cross‐sectional views of g) gelatin film and gelatin/charcoal composite films with h) 10, i) 15, j) 20, k) 25, and l) 30 wt% charcoal. Scale bars represent 50 µm.

**Figure** **2**a displays the scanning electron microscopy (SEM) micrograph of charcoal particles, revealing lateral particle sizes ranging from several microns to tens of microns. Figure 2b shows the statistical distribution of particle sizes, with an average of 6.9 ± 6.6 µm, indicating that the particles are predominantly in the microscale range. Figure 2c–f illustrate the morphology of the top and bottom surfaces of pure gelatin films and gelatin/charcoal composite films with 10 wt% filler. Both surfaces of the gelatin film are smooth, whereas the upper surface of the gelatin/charcoal film shows a slightly textured appearance, and the lower surface is densely packed with charcoal particles. Results for other filler contents are provided in Figure S1 in the Supporting Information (SI), showing similar surface morphology across all films. These observations suggest phase separation in the gelatin/charcoal films, with gelatin molecules remaining at the top and charcoal particles concentrating at the bottom as a result of sedimentation, upon samples’ drying. This distinct phase separation is further confirmed by the cross‐sectional images in Figure 2g‐l, which also show that higher filler content leads to the increase of the thickness of the charcoal‐rich layer, and to improved electrical conductivity, as will be discussed in detail later.

**Figure 3:**
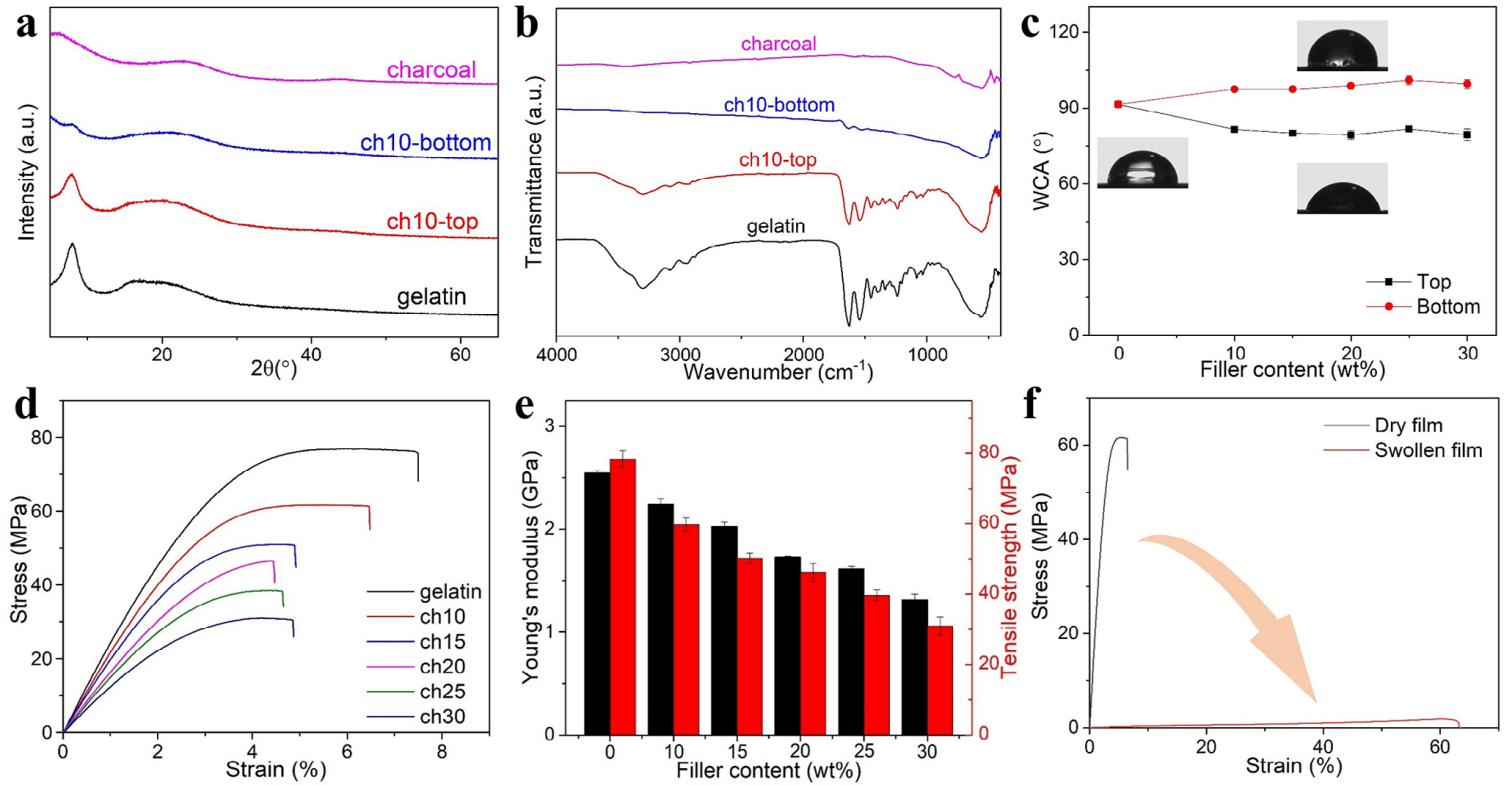
a) XRD and b) FTIR analyses of charcoal particles, gelatin, and gelatin/charcoal composite films. c) Water contact angle (WCA) measurements for gelatin and gelatin/charcoal films. d) Stress–strain curves for gelatin and gelatin/charcoal films. e) Changes in Young's modulus and tensile strength of the films with varying filler content. f) Stress‐strain curves of dry and swollen films.

The crystalline and chemical structures of the charcoal particles and gelatin‐based films were analyzed using X‐ray diffraction (XRD) and Fourier transform infrared (FTIR) spectroscopy, as shown in **Figure** **3**a,b. The bottom layer of the composite film exhibits structural similarities with charcoal particles, while the top layer resembles gelatin, further supporting evidence of phase separation within the composite. In addition, the charcoal used did not contain oxygen‐containing functional groups, and its incorporation did not alter the chemical structure of gelatin. This suggests that the interactions between charcoal and gelatin are primarily physical or surface‐based, rather than involving strong chemical bonding. Figure 3c demonstrates how phase separation affects wettability, with the top layers displaying contact angles between 79.3° and 81.8°, indicating hydrophilic properties, and the bottom layers showing moderately hydrophobic characteristics, with contact angles ranging from 97.5 ± 0.5° to 101.0 ± 1.7°. The stress‐strain curves for pure gelatin and its composites with varying AC loadings are shown in Figure 3d, revealing that the Young's modulus, tensile strength, and elongation at break decrease as charcoal content increases. Figure 3e highlights the changes in Young's modulus and tensile strength with different charcoal contents; specifically, with 10 wt% charcoal, the Young's modulus decreases slightly from 2.5 to 2.2 GPa, and the tensile strength drops from 78.3 to 60.0 MPa. The phase separation between the gelatin and charcoal introduces weak interfacial regions where load transfer is inefficient, leading to a reduction of the mechanical strength and modulus of the composite. These weak interfaces become points of mechanical failure under stress. Despite the reduction in mechanical properties, the 10 wt% charcoal film maintains a notable mechanical strength, successfully lifting a 500 g weight, as shown in Figure S2 (SI). Since the edible sensor is intended to function under GI conditions rich in fluids, the swollen‐state mechanical properties of the film were evaluated as shown in Figure 3f. The tensile strain reached approximately 60% in the swollen state, which is sufficient to accommodate and monitor the dynamic movements of the GI tract—for example, stomach expansion typically involves strains of approximately 30–60%.

### 2.2 Strain Sensing

**Figure 4:**
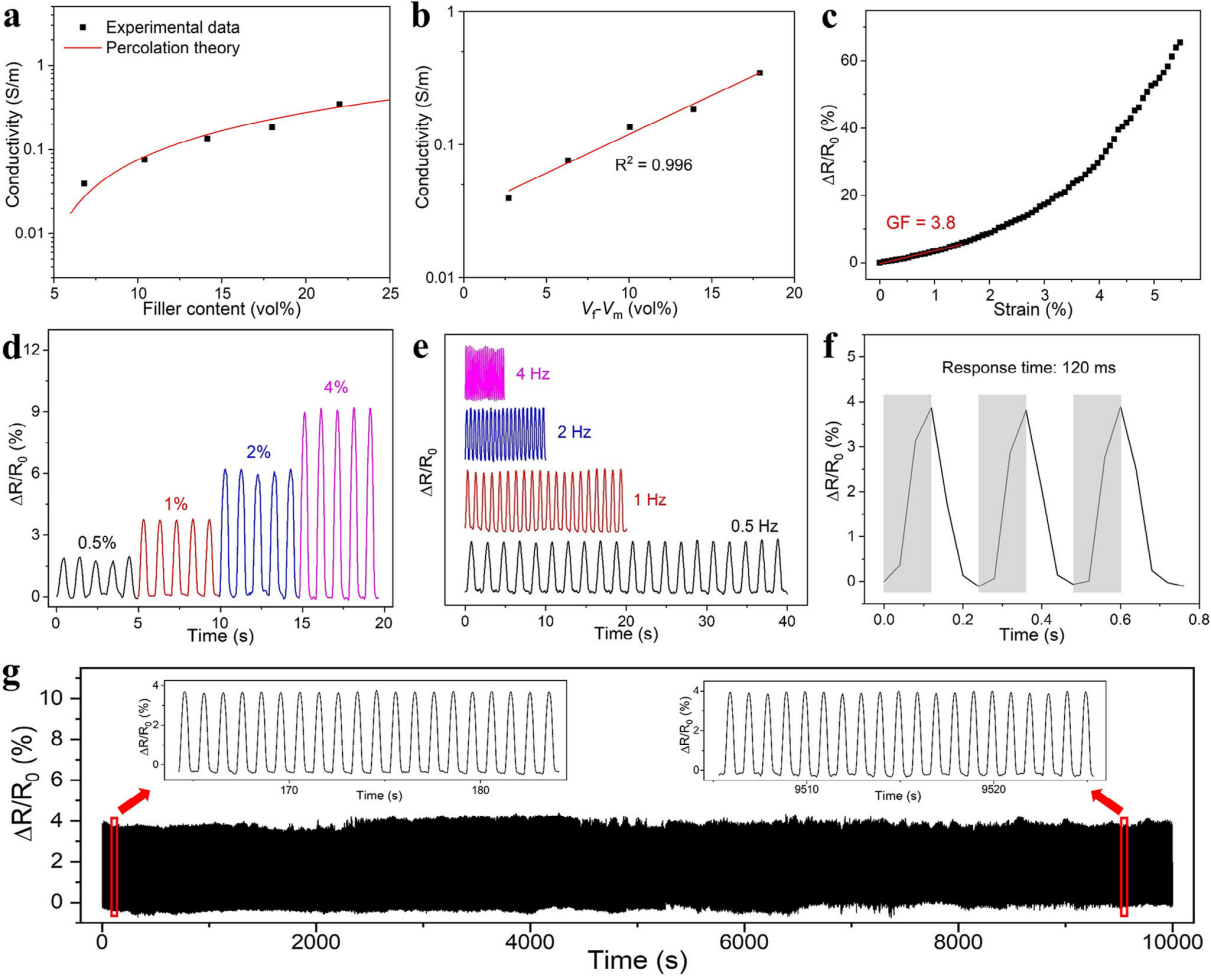
a) Electrical conductivity and percolation fit of gelatin/charcoal films. b) Linear log–log plot of conductivity versus volume fraction. c) Change in normalized resistance with applied strain for the gelatin/charcoal film (10 wt% filler). d) Dynamic strain sensing of the film at 0.5, 1, 2, and 4% strain (1 Hz). e) Dynamic strain sensing of the film at 0.5, 1, 2, and 4 Hz (1% strain). f) Film response time at 4 Hz. g) Dynamic strain sensing performance of the film over 10000 cycles (1% strain at 1 Hz).

As shown in **Figure** **4**a, the addition of AC particles imparts electrical conductivity to the gelatin films, with conductivity increasing as the volume fraction of filler increases. The conversion of AC weight content to volume fraction is detailed in Section S3 (SI). The edible film achieves an electrical conductivity of 0.04 S m^−1^ with 6.82 vol% filler and 0.35 S m^−1^ with 22.01 vol% filler. The measured electrical conductivity values are comparable to those reported in previous studies using activated carbon as an edible electrode (conductivity range of 0.1–200 S m^−1^).^[^ ^25^ ^]^ Nevertheless, this work offers the added advantage of a flexible material with the potential for large‐scale production, making it compatible with composite industry practices and equipment.

The variation of electrical conductivity with filler content can be fitted by the percolation theory as σ  = σ_0_ (*V*_f_ − *V*_c_)^*t*^,^[^ ^36^ ^]^ where *σ* and *σ*_0_ are the conductivities of the composite and filler, *V*_f_ is the volume fraction of the filler, *V*_c_ is the percolation threshold and *t* is the percolation exponent. For the fitting, the parameters *σ*_0_ = 0.0135 S m^−1^, *V*_c_ = 4.1 vol% and *t* = 1.3 were used. The percolation exponent, *t*, typically has a value of ≈1.33 for 2D systems and ≈2 for 3D systems.^[^ ^36^ ^]^ The value of 1.3 indicates that a 2D conductive network was formed within the film, induced by the assembly of charcoal particles at the bottom of the film as revealed from the SEM images. The percolation threshold, at 4.1 vol%, is relatively high due to the charcoal particles’ shape, which resembles that of carbon black and lacks a distinct aspect ratio, and is lower that all the concentrations used in this work.^[^ ^37^ ^]^ Based on this threshold, the linear fit in Figure 4b shows a high accuracy in conductivity fitting, with a coefficient of determination of 0.996. The composite film with 10 wt% charcoal (6.82 vol%), which demonstrates a good balance between mechanical strength and electrical conductivity, is selected for subsequent electromechanical and sensing applications. As illustrated in Figure 4c, stretching the composite film results in an increase in electrical resistance corresponding to the applied strain. As the film was strained, the conductive pathways within the edible composite material were elongated, resulting in less contact between the fillers and therefore an increase in resistance. This resistance change was directly correlated with the magnitude of applied strain, enabling real‐time monitoring. Following the initial linear stage between resistance and strain, the gauge factor (*G*) can be derived by fitting the experimental data as Δ*R*/*R*_0_ =  *G*ε, where Δ*R*/*R*_0_ is the change of electrical resistance and *ε* is the applied strain. The working factor, *W*, is the strain limit at which the relative resistance change is no longer linear with strain.^[^ ^38^ ^]^ The parameters of *W* = 1.5% and *G* = 3.8 were determined from the fitting in Figure 4c.

The dynamic strain sensing behavior of the film under varying strains, frequencies, and cycles is depicted in Figure 4d–g. As shown in Figure 4d, the relative resistance increases progressively with strain increments from 0.5% to 4%, and there is minimal variation in resistance at a given strain level, indicating that the film provides precise strain detection and consistent repeatability across different strain levels. Additionally, as illustrated in Figure 4e, the resistance signals remain stable as the frequency increases from 1 to 4 Hz, suggesting the film's capability to operate accurately at higher frequencies. Figure 4f presents the film's response time under 4 Hz and 1% strain, which is approximately 120 ms—sufficient for applications such as human movement and pulse monitoring. The film's fatigue resistance, tested at 1% strain and 1 Hz for up to 10 000 cycles, is shown in Figure 4g. The resistance signals remain highly stable over the entire test, unaffected by the increase in cycles. Insets reveal that the initial and final signals closely match, underscoring the gelatin/charcoal film's excellent dynamic sensing stability and fatigue resistance.

### 2.3 Body Movement Monitoring and Writing Recognition

**Figure 5:**
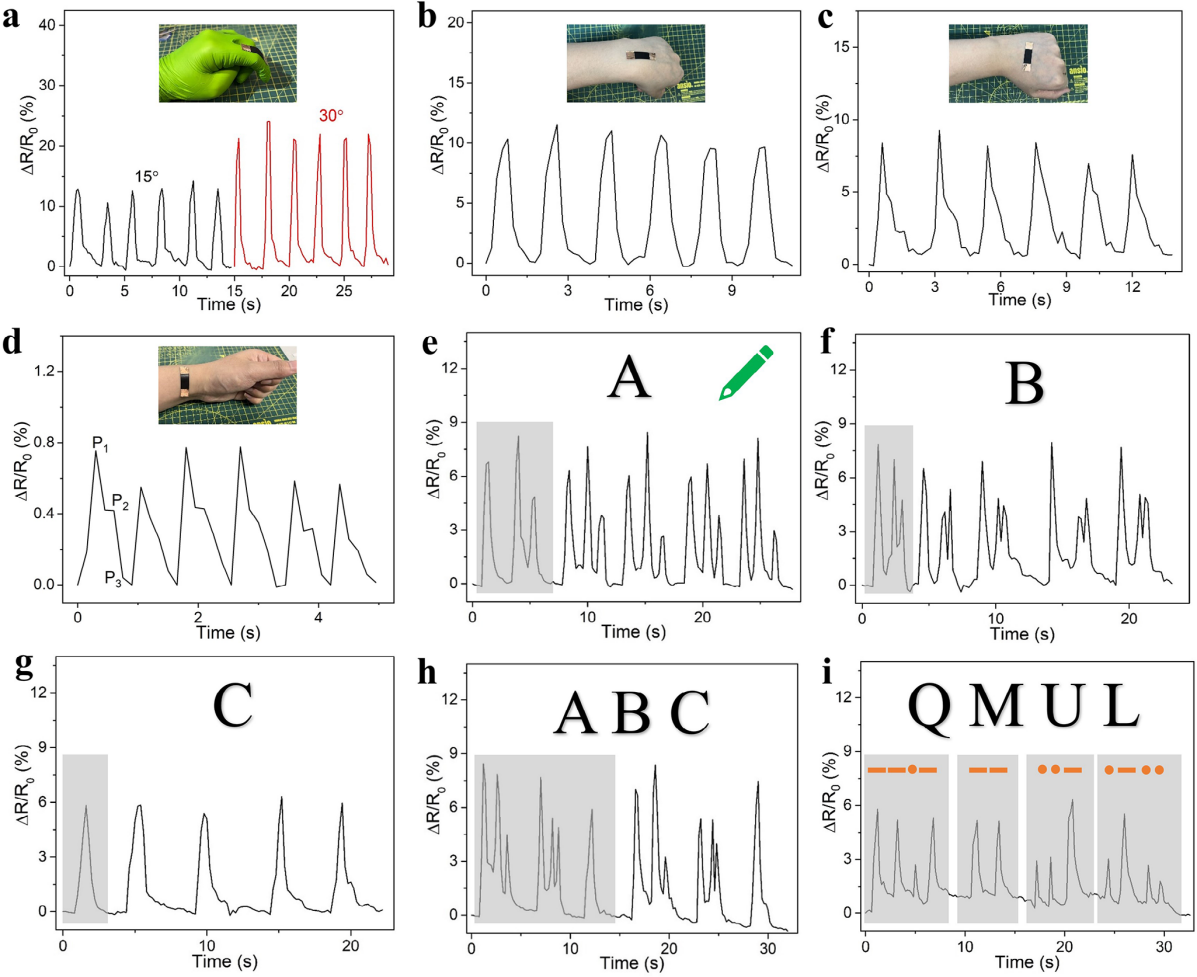
The application of the gelatin/charcoal film for body movement monitoring, including a) finger bending, b) writing bending and c) fist holding, and d) pulse monitoring. The application of the gelatin/charcoal film for writing recognition, including e) “A,” f) “B,” g) “C,” h) “ABC” and i) Morse code “QMUL” (一一·一, 一一, ··一, ·一··).

Given its reliable electromechanical signals and strong fatigue resistance, the edible film with 10 wt% filler content can be used for tracking body movements and handwriting recognition. When attached to the knuckle (the top, gelatin surface is in direct contact with the knuckle), the film can detect finger bending and stretching in real time, producing steady, periodic signals (**Figure** **5**a). The resistance variation increases with minimal hysteresis with the bending angle, demonstrating the film's accuracy in monitoring finger movements. It's worth noting that due to its limited elongation at break (≈6.2%), the film is best suited for tracking movements with small amplitudes. When positioned on the wrist or back of the hand, the film can also detect wrist bending (Figure 5b) and fist clenching (Figure 5c). These motions, being of smaller amplitude than finger bending, result in smaller resistance changes. The composite film can monitor not only physical movements but also subtle physiological signals, such as pulse. As shown in Figure 5d, wrist pulse waveforms were recorded with the film adhered to the radial artery. The resistance variations were significantly smaller than those from bodily movements, and three characteristic peaks – percussion wave (P1), tidal wave (P2), and dicrotic wave (P3)—were observed, indicating the film's high sensitivity and rapid response.

Beyond body movement monitoring, the fabricated gelatin/charcoal film is also suitable for writing recognition, thanks to its combined mechanical and electrical properties. When writing on the film (the bottom, gelatin/AC composite surface is in direct contact with the pen), variations in style ‐such as order, pressure, and speed‐ generate unique resistance signals, enabling the recognition of written words. Figure 5e–g demonstrate that the electrical signal for the letter “A” shows two strong peaks and one weak peak, “B” produces one strong peak followed by two connected peaks, and “C” displays a single peak. These distinctive signal patterns allow for the clear recognition of individual letters. Furthermore, across five repeated cycles, the signals for each letter show consistent repeatability, with only minor variations in intensity due to handwriting pressure. Additionally, when the letters “ABC” are written sequentially, each letter's signal aligns with its individual counterpart (Figure 5h). The film is also capable of interpreting Morse code, as demonstrated by the accurate identification of “QMUL” in Figure 5i. In this example, dashes are represented by greater amplitude and duration compared to dots. Other Morse code signals, such as “SOS” and “2024,” also exhibit clear, distinguishing features due to the unique combinations of dots and dashes (see Figure S3, SI). This demonstrates the potential of the gelatin/charcoal film as a writing recognition electronic device.

### 2.4 Humidity Sensing and Human Respiration Monitoring

**Figure 6:**
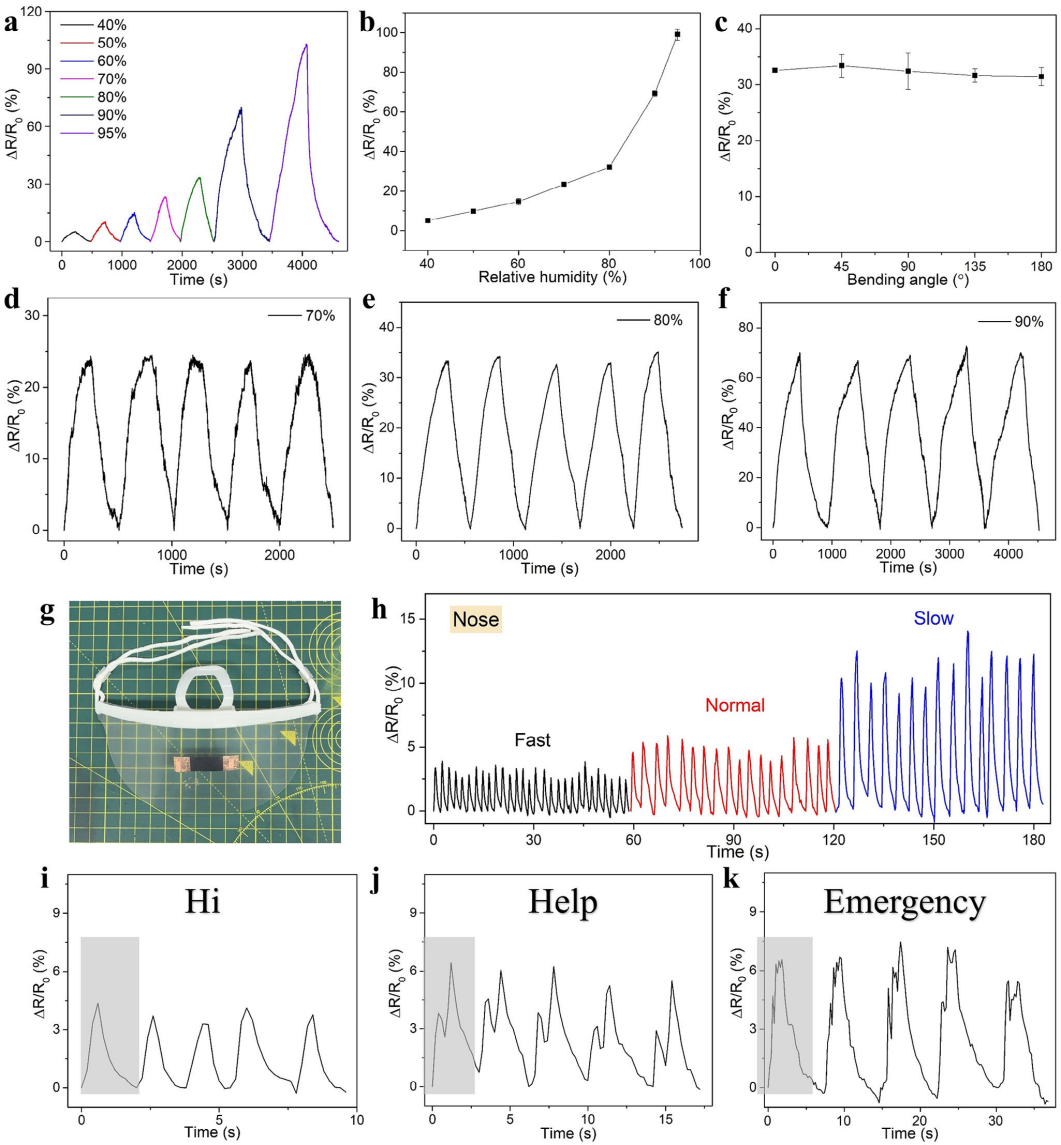
a) Variation of normalized resistance of the gelatin/charcoal film (10 wt% filler) across different humidity levels. b) Statistical analysis of response variations versus relative humidity. c) Normalized resistance variation at different bending angles. Dynamic response and recovery of the film at d) 70%, e) 80%, and f) 90% relative humidity. g) Film attached to a mask for respiration monitoring. h) Response curve of the film to nose breathing at varying respiratory rates. Response curves of the film during speech, including i) “Hi,” j) “Help,” and k) “Emergency”.

Humidity sensing is crucial for both environmental monitoring and personal health management.^[^ ^13^, ^15^ ^]^ Environmental humidity plays a vital role in soil and atmospheric monitoring, as well as in various industrial manufacturing processes. Additionally, the humidity in exhaled breath provides valuable insights into the intensity and speed of respiration, serving as a potential indicator of lung health and the presence of infections. In this section, we explore the humidity sensing potential of our edible gelatin/charcoal film. **Figure** **6**a shows the film's resistance response to varying humidity levels from 40% to 95%. The film's resistance is sensitive to changes in humidity, with the degree of resistance variation increasing as humidity levels rise. At 70% humidity, the resistance changes by 23.4%, reaching a peak change of 102.2% at 95% humidity. This indicates that at higher humidity levels, the film absorbs more water molecules, resulting in moderate swelling of the film. This leads to a reduction of the effective contact between charcoal particles and increases the resistance of the film. Figure 6b presents the statistical analysis of the resistance response, showing a linear resistance variation below 80% humidity, with a significant increase above 90%. Figure 6c examines the film's sensing response at 80% humidity under different bending angles from 0° (flat) to 180° (folded), showing no noticeable change in humidity response, which highlights the film's robustness against bending and folding. Figure 6d–f demonstrate the film's cyclic moisture absorption and desorption recovery at humidity levels of 70%, 80%, and 90%, with consistent resistance change rates over five cycles, showcasing the excellent repeatability of the gelatin/charcoal humidity sensor.

As shown in Figure 6g, the gelatin/charcoal film was attached to a face mask for respiration monitoring due to its high sensitivity and repeatability. The film's resistance varies with each breath, allowing the tracking of nasal breathing frequency by counting the number of peaks, as illustrated in Figure 6h. The observed breathing rates are approximately 29, 18, and 15 breaths per minute for fast, normal, and slow breathing, respectively. As the breathing rate slows, more moisture is absorbed, resulting in wider and taller waveforms. The gelatin/charcoal film can not only detect the breathing frequency but also the type of breathing. When used to monitor mouth breathing, the film exhibits a greater change in resistance (Figure S4, SI), likely due to the higher moisture release associated with mouth breathing compared to nasal breathing. This capability allows the film to distinguish between different types of respiration. Figure 6i–k illustrate a schematic of the film detecting exhaled air during speech. Distinct peaks correspond to each spoken syllable, with “Hi” (a monosyllabic word) producing a single peak (Figure 6i), “Help” (a bisyllabic word) generating two peaks (Figure 6j), and “Emergency” (a multisyllabic word) resulting in multiple peaks (Figure 6k).

### 2.5 Temperature Sensing

**Figure 7:**
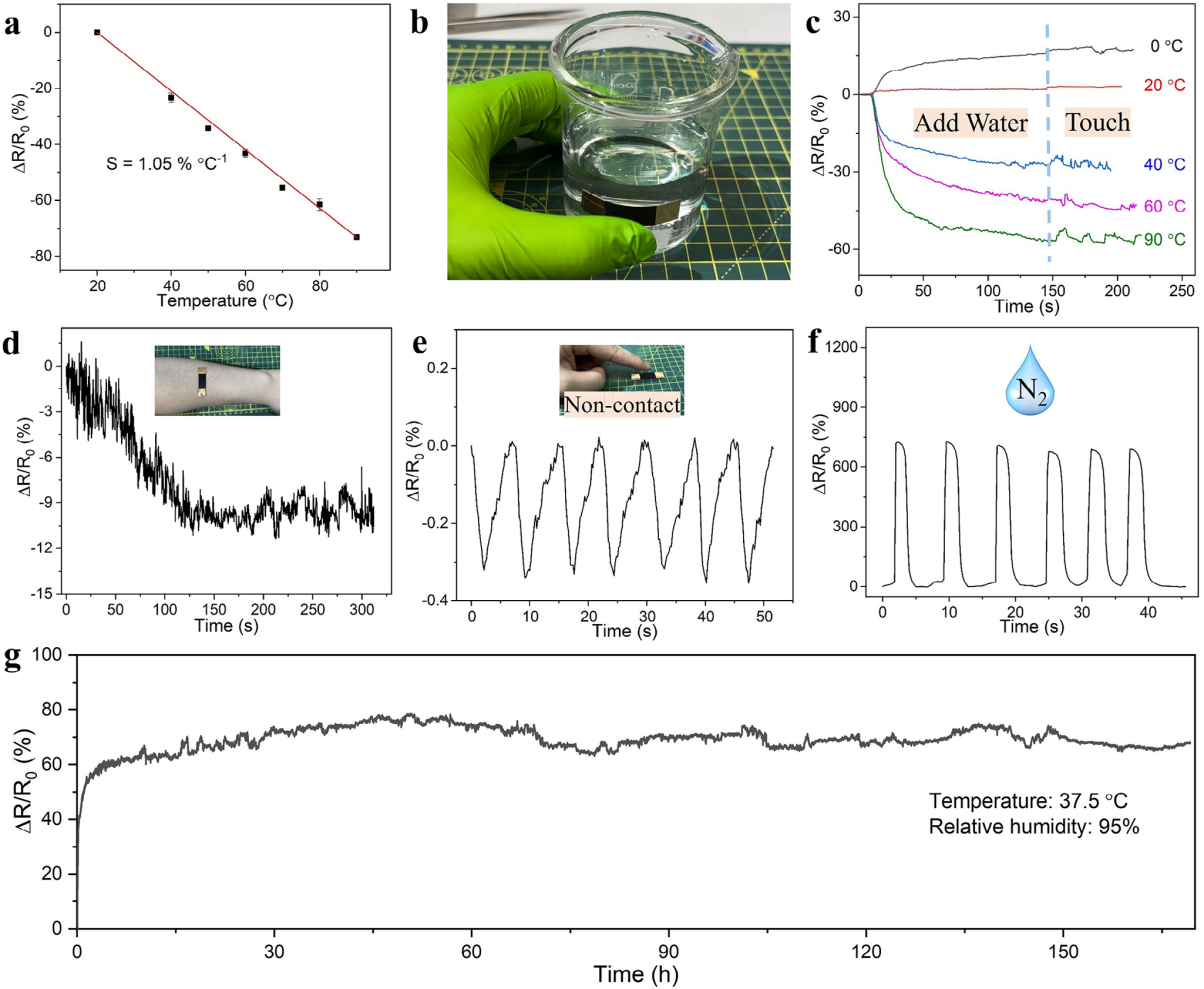
a) Relative resistance change of the gelatin/charcoal film (10 wt% filler) for temperature sensing. b) Film attached to a beaker for water temperature detection. c) Relative resistance change of the film for detecting water temperature and fingertip contact. Applications of the film for sensing d) body temperature, e) fingertip temperature (non‐contact), and f) liquid nitrogen exposure. g) Working stability of the film in a simulated gastric environment with a temperature of 37.5 °C and relative humidity of 95% for a week.

In addition to detecting strain and humidity, the edible film also responds to temperature changes.^[^ ^39^ ^]^ As shown in **Figure** **7**a, the film's resistance decreases as temperature increases, with a 23.3% reduction at 40 °C and a 73.1% reduction at 90 °C. A linear fit of resistance variation with temperature yields a slope of 1.05%°C−^1^, indicating high temperature sensitivity, which surpasses previously reported values (0.6–1.0%°C−^1^ for paper‐based sensor^[^ ^40^ ^]^). The resistance variation as a function of temperature in this system is likely dominated by the thermal activation of electrons, characteristic of a semiconducting behavior attributed from the activated charcoal particles.^[^ ^41^ ^]^ As the temperature rises, more charge carriers are thermally excited into the conduction band, reducing the electrical resistance. This reduction offsets the increase in resistance caused by electron‐phonon scattering at higher temperatures, resulting in an overall decrease in electrical resistance with increasing temperature.

Figure 7b illustrates the film's application in sensing water at different temperatures (while being adhered to the external side of a glass beaker) and subsequently sensing a finger touch, with corresponding resistance variations displayed in Figure 7c. When room temperature (20 °C) water is introduced into the glass beaker, the film's resistance remains stable, reflecting its reliability. The resistance decreases with increasingly hot water (40, 60, and 90 °C), aligning with the temperature increase. Conversely, the resistance increases with cold water (0 °C), suggesting the film's capability to detect temperatures below room temperature. Touching the film induces resistance fluctuations, due to pressure changes. Our findings indicate that the film can differentiate between various stimuli, supporting its use in multimodal sensing applications.

The film can detect not only significant temperature shifts but also subtle ones. For instance, it can measure body temperature, as shown in Figure 7d, where its resistance gradually decreases over time upon attachment to the body, before stabilizing at a relatively constant value. This behavior demonstrates the sensor's sensitivity to temperature changes. Such a response confirms the sensor's capability for continuous, non‐invasive monitoring of body temperature through resistance‐based readouts. Additionally, the film is suitable for non‐contact sensing applications, as illustrated in Figure 7e. When a fingertip approaches the film (≈1 mm distance), the resistance decreases due to the localized increase in temperature. Upon moving the fingertip away, the resistance returns to its original level. The film demonstrates good repeatability in its response to forward and backward fingertip movements, suggesting potential for non‐contact sensing, which could help reduce virus transmission risks.

Furthermore, the film demonstrates exceptional sensitivity to extremely low temperatures. As shown in Figure 7f, the application of liquid nitrogen causes a dramatic increase in resistance (≈700%), which quickly returns to baseline within 3–4 s. These measurements highlight the film's high reproducibility and stability under repeated exposure to liquid nitrogen. Overall, the film exhibits outstanding temperature detection capabilities, effectively operating across a wide range, from elevated to extremely low temperatures. Figure 7g illustrates the sensing stability of the film under a simulated gastric environment. The film demonstrated stable sensing performance under simulated gastric conditions for a week, indicating its potential suitability for in vivo applications such as short‐term GI monitoring or diagnostics, where stability in harsh internal environments is essential.

### 2.6 Biodegradability and Recyclability

**Figure 8:**
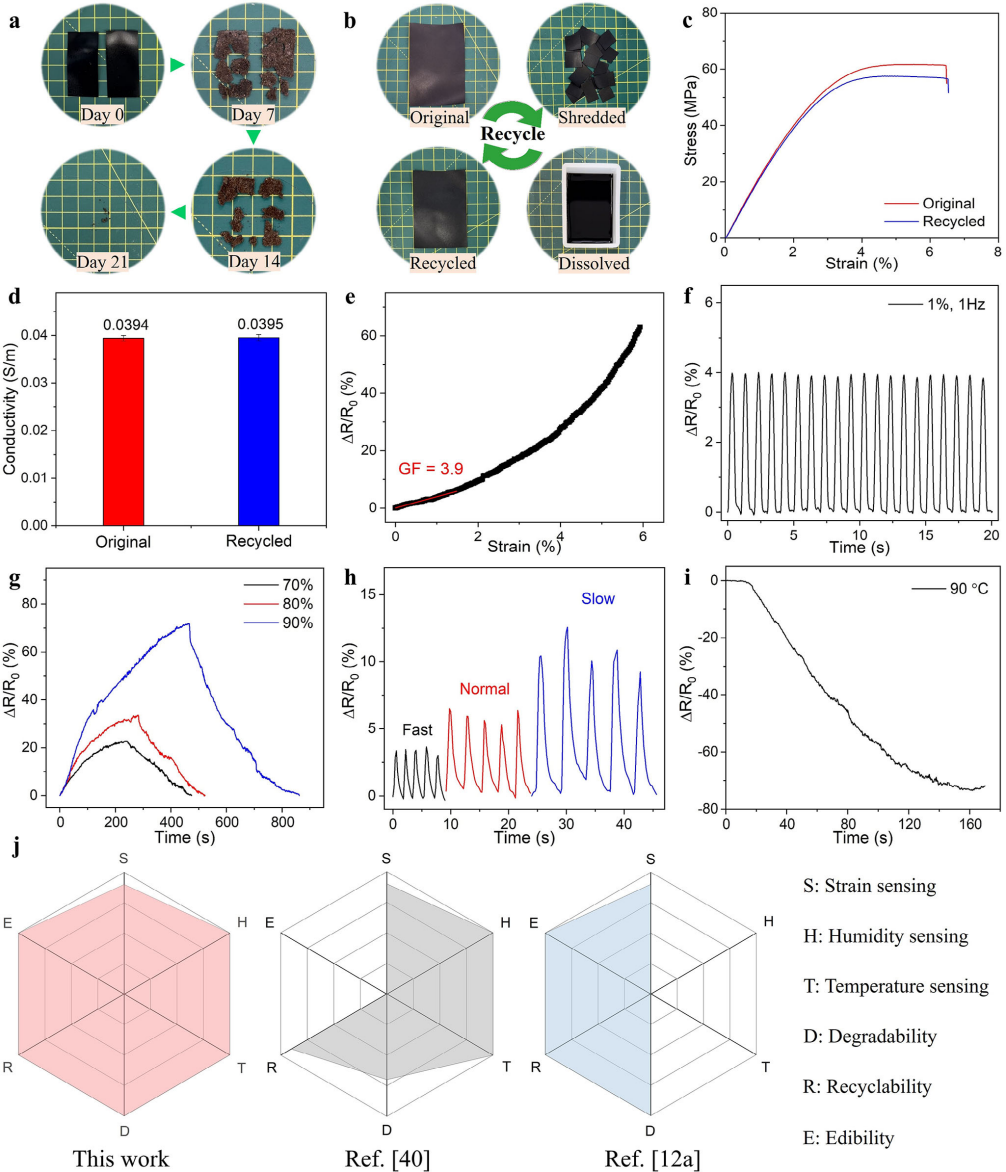
a) Images of the edible film after burial in natural soil. b) Images showing the recycling process of the edible film. c) Stress‐strain curve, d) electrical conductivity, e) electromechanical response, f) dynamic strain sensing, g) humidity sensing response, h) respiration monitoring, and i) temperature sensing response of the recycled film. j) Radar plots comprehensively comparing the performance of the fabricated edible multimodal sensor with conventional multimodal sensor^[^ ^40^ ^]^ and edible strain sensor.^[^ ^12a^ ^]^

A large number of electronic devices today are made from non‐biodegradable materials, contributing to a considerable buildup of e‐waste.^[^ ^10a^ ^]^ The evolution of biodegradable electronics, particularly edible ones, presents a promising approach to addressing e‐waste accumulation. Additionally, incorporating recyclability into these devices further enhances their sustainability, ensuring that valuable resources can be recovered and reused, reducing the demand for raw materials, and creating a more circular and resource‐efficient technology lifecycle. To assess the environmental degradation of the edible film, a soil burial test was conducted (**Figure** **8**a). The results show that the film degraded rapidly after burial, breaking into small fragments within 7 d, with most of the film decomposed by day 14. Notably, the film was disintegrated after 21 d, indicating active microbial breakdown and degradation of the edible film.^[^ ^42^ ^]^ Since gelatin and charcoal are both natural materials, the film's disintegration is not ecotoxic, especially considering the low amount of AC used. This sustainable composition ensures that as the film decomposes, it seamlessly integrates back into the environment without contributing to pollution. Very importantly, the film is not only edible and biodegradable but also recyclable. Typically, recycling processes often lead to a reduction in electromechanical performance; however, this film defies that trend by maintaining its properties after recycling. As shown in Figure 8b, the original film can be easily recycled into a new film by shredding, dissolving in water, and casting into a mold. The dissolving process takes under thirty minutes, highlighting the film's excellent processability and eco‐friendliness. The mechanical, electrical, electromechanical, and sensing properties of the recycled film were assessed (Figure 8c–i). The recycled film demonstrates comparable tensile strength (Figure 8c), electrical conductivity (Figure 8d), and gauge factor (Figure 8e) to the original, indicating that the recycling process does not diminish its properties. Additionally, the recycled film performs excellently in multimodal sensing applications, including strain sensing (Figure 8f), humidity sensing (Figure 8g), respiration monitoring (Figure 8h), and temperature sensing (Figure 8i).

Figure 8j demonstrates that the developed multimodal sensors possess benefits of both edibility and degradability over conventional multimodal sensors,^[^ ^40^ ^]^ as well as multimodal sensing capabilities beyond those of existing edible sensors.^[^ ^12a^ ^]^ These results reveal the film's unique blend of edibility, biodegradability, recyclability, and multimodal sensing, setting it apart as a breakthrough in sustainable electronics. Its ability to retain high functionality after recycling, positions it as an environmentally responsible material capable of high‐precision sensing and monitoring, offering a practical and impactful step towards a more sustainable future.

## 3 Conclusions

In pursuit of materials that can be utilized for both edible electronics and environmental monitoring, we have developed electrically conductive composite films using gelatin and activated charcoal. Both the gelatin matrix and charcoal particles are safe for ingestion and can be processed in water, with the composite films produced through a simple, sustainable and highly scalable one‐step method. The resulting bi‐layer films have tunable mechanical and electrical properties, achieved by varying AC content. We demonstrate the efficient use of the edible composite film with 10 wt% AC for high‐precision multimodal sensing. For strain sensing, the material shows high sensitivity, fast response, and excellent fatigue resistance, making it suitable for applications such as in body movement tracking and handwriting recognition. For humidity sensing, the film has a broad detection range and good repeatability, enabling applications such as respiration monitoring and speech detection. In temperature sensing, the film covers a wide temperature range, representative of standard environmental conditions, and is effective for body temperature detection, non‐contact sensing, and liquid nitrogen detection.

Quite importantly, the materials presented herein, can disintegrate in the environment without creating harmful byproducts, considering the limited amount of AC used per device (Table S1, SI) and the biodegradable nature of gelatin. The recycled film retains almost identical mechanical, electrical, and sensing properties to the original, proving its robustness. Our study not only showcases the potential of edible electronics to reduce e‐waste but also contributes significantly to the advancement of green, eco‐friendly technologies for future sensing applications.

## 4 Experimental Section

### Materials

Activated charcoal (Supelco, powder) was provided by Sigma‐Aldrich. Gluten‐free beef gelatin was purchased from Mr.P Ingredients.

### Preparation of Gelatin/Charcoal Composite Films

The preparation process for the gelatin/charcoal composite films is shown in Figure 1. Initially, a specified amount of charcoal was dispersed in DI water using probe sonication at 40% amplitude, with a cycle of 3 s on and 2 s off for 5 min. Gelatin was then added to the charcoal suspension, and the mixture was stirred at 60 °C for 30 min to achieve a uniform dispersion. The resulting mixture was cast into a silicone mold and allowed to dry at room temperature for ≈4 d. Pure gelatin films, along with gelatin/charcoal composite films containing 10, 15, 20, 25, and 30 wt% charcoal, were prepared. Both gelatin and charcoal are food‐grade materials that can be safely consumed in substantial amounts. The quantitative analysis of the edibility of the composite films with 10 wt% charcoal is illustrated in Table S1 in the SI. The amount of gelatin (36 mg) and charcoal (4 mg) in each sensor film (4 × 1 cm^2^) is far below than the amount can be taken daily (10 g and hundreds of mg, respectively^[^ ^43^ ^]^), ensuring the composite films are non‐toxic and safe to ingest. Additionally, the use of DI water that is arguably the most eco‐friendly solvent, in the mixing process, eliminates the risk of toxic solvent residues.

### Characterization

The morphology of charcoal particles, gelatin, and gelatin/charcoal films was analyzed using SEM (FEI Inspect‐F, Netherlands) at an operating voltage of 5 kV. Crystal characteristics were assessed via XRD (ANalytical X'Pert‐Pro), and the molecular structures were examined using FTIR (TENSOR 27) in transmission mode. The WCA of the films was measured with a Contact Angle DSA 100. Tensile properties were tested on an Instron 68TM‐10 equipped with a 2 kN static load cell and a loading rate of 1 mm min^−1^. Electrical conductivity measurements were performed using the Keysight DAQ970A Data Acquisition System.

For multimodal sensing applications, two copper tapes were attached to the two ends of the film strip (4 × 1 cm^2^) and its resistance was recorded with a Keysight DAQ970A Data Acquisition System. Strain sensing under static and dynamic loads was conducted using Instron 68TM‐10 and Instron E1000, respectively. For body motion monitoring, the film strip was attached to various body parts, and for handwriting recognition, it was adhered to a silicone substrate. All bodily testing was performed by the first author, Ming Dong, on himself, and he provided written informed consent for the procedures. For humidity sensing, the film strip was placed in an environmental chamber at a constant temperature of 25 °C with relative humidity levels ranging from 40% to 95%. During the measurement, the humidity increased from the baseline level (≈30%) to the intended humidity level and then returned to the baseline level, resulting in a characteristic sawtooth profile. The film strip was also attached to a mask for respiration monitoring. For temperature sensing, it was initially affixed to a hot plate with temperatures varying from 40 °C to 90 °C. For hot water sensing, the sample was adhered to the outer surface of a glass beaker, into which water at different temperatures was poured. The relative resistance was then recorded as a function of time and water temperature. Finally, the gastric environment was simulated by maintaining a temperature of 37.5 °C and a relative humidity of 95% in an environmental chamber, providing a simplified model to assess sensor stability under physiologically relevant conditions.

Biodegradability was assessed via soil burial, with the film strip buried at a 3 cm depth in natural soil, and digital images were taken periodically. For recyclability, the original film was shredded, dissolved in DI water, and recast in a silicone mold. Once dried, the properties of the recycled film were evaluated.

## Conflict of Interest

The authors declare no conflict of interest.

## Supporting information



Supporting Information

## Data Availability

The data that support the findings of this study are available from the corresponding author upon reasonable request.
