# Supplementary material for: Edible and Recyclable Gelatin‐Based Electronics for High‐Precision Health and Environmental Monitoring
Source: Adv Sci (Weinh). 2025 Aug 4;13(15):e07950. doi: 10.1002/advs.202507950 (PMC13042497; doi:10.1002/advs.202507950)
Supplement: Supplementary file 1 — Supporting Information [file ADVS-13-e07950-s001.docx]

**Edible and Recyclable Gelatin-based Electronics for High-Precision Health and Environmental Monitoring**

**Ming Dong^1,*^, Pietro Cataldi^2^, Han Zhang^3^, Emiliano Bilotti^4^, Conor Boland^5^, Athanassia Athanassiou^2^, Dimitrios G. Papageorgiou^1,*^**

*^1^ School of Engineering and Materials Science, Queen Mary University of London, London E1 4NS, United Kingdom*

*^2^ Smart Materials Laboratory, Istituto Italiano di Tecnologia, Via Morego 30, Genova 16163, Italy*

*^3^WMG, University of Warwick, Coventry, CV4 7AL, UK*

*^4^Department of Aeronautics, Imperial College London, Exhibition Road, London SW7 2AZ, UK*

*^5^ School of Mechanical and Manufacturing Engineering, Dublin City University, Glasnevin, Dublin 9, Ireland*

******Corresponding authors′ emails:* [*ming.dong@qmul.ac.uk*](mailto:ming.dong@qmul.ac.uk)*;* [*d.papageorgiou@qmul.ac.uk*](mailto:d.papageorgiou@qmul.ac.uk)

**SUPPORTING INFORMATION**

**Contents:**

**S1. SEM images of gelatin/charcoal films**

**S2. Conversion of weight content to volume fraction**

**S3. Lifting a 500 g weight using the edible film**

**S4. Morse codes of ‘SOS’ and ‘2024’**

**S5. Mouth breathing monitoring**

**S6. Edibility of gelatin/charcoal ingredients**

**S1. SEM images of gelatin/charcoal films**


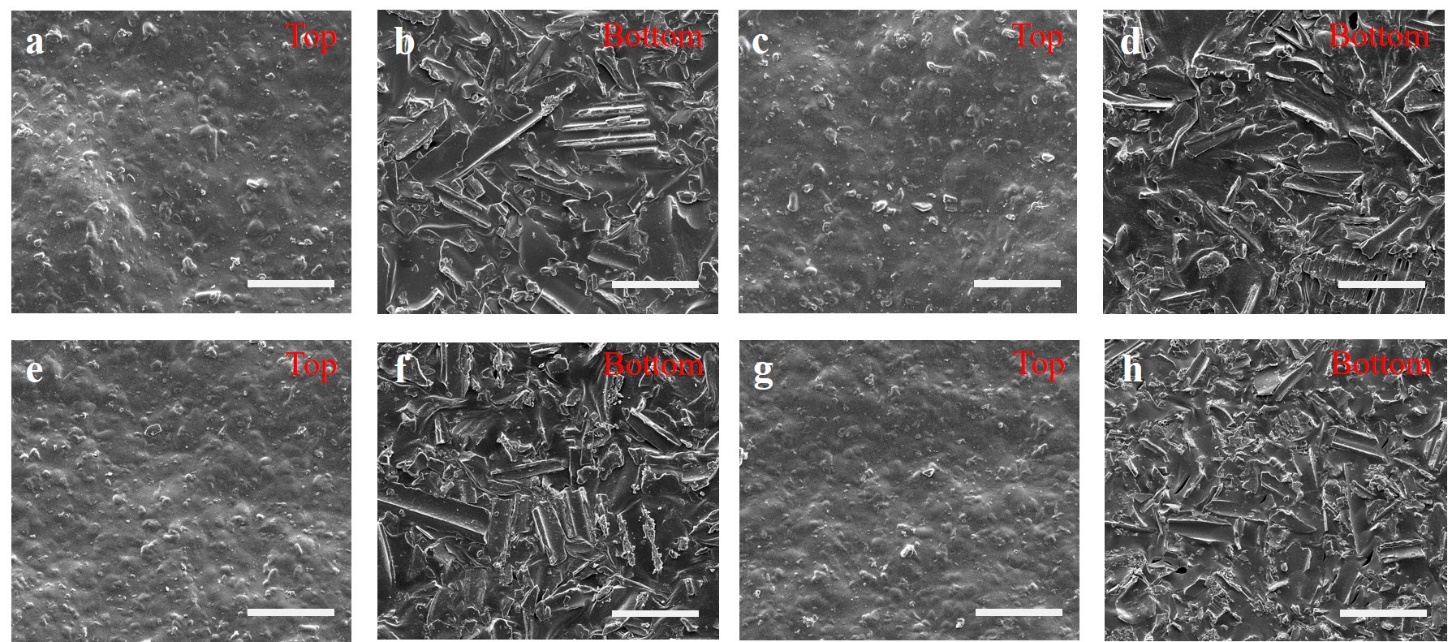


Figure S1. a) Top and b) bottom surfaces of the gelatin/charcoal film with 15 wt%, c, d) 20 wt%, e, f) 25 wt% and g, h) 30 wt% charcoal. Scale bars, 30 μm.

**S2. Conversion of weight content to volume fraction**

The weight ratio of charcoal particles was converted to volume fraction using the following equation as

|  | $V_{f}=\frac{w_{f}\rho_{m}}{w_{f}\rho_{m}+\left( 1-w_{f} \right)\rho_{f}},$ | (s1) |
| --- | --- | --- |

where *V*_f_ and *w*_f_ are the volume fraction and the weight fraction of the filler, and *ρ*_m_ and *ρ*_f_ are the density of the matrix and the filler, respectively. The density of potato starch is 1.35 g/cm^3^ and the density of charcoal is 2.05 g/cm^3^. Then, the weight ratios of 10, 15, 20, 25 and 30 wt% were converted to volume fractions of 6.82, 10.41, 14.14, 18.00 and 17.61 vol%, respectively.

**S3. Lifting a 500 g weight using the edible film**


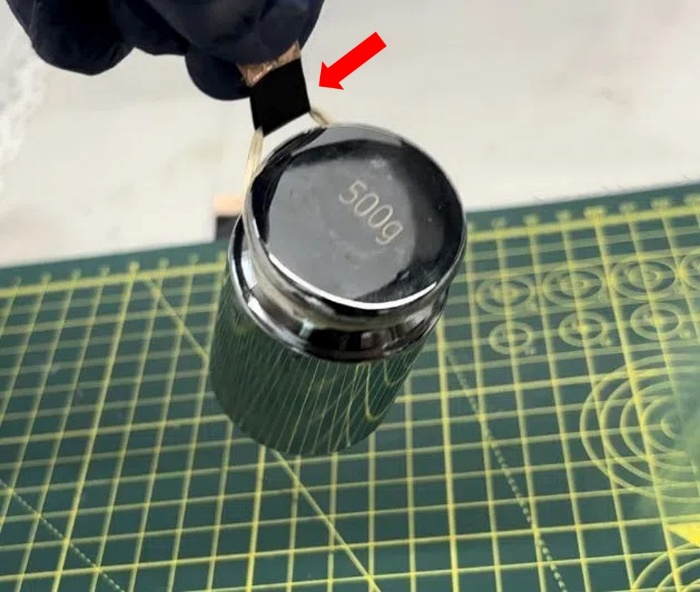


Figure S2. Demonstration of the gelatin/charcoal film strip (indicated with a red arrow - 10 wt% AC) lifting a 500 g weight.

**S4. Morse codes of ‘SOS’ and ‘2024’**


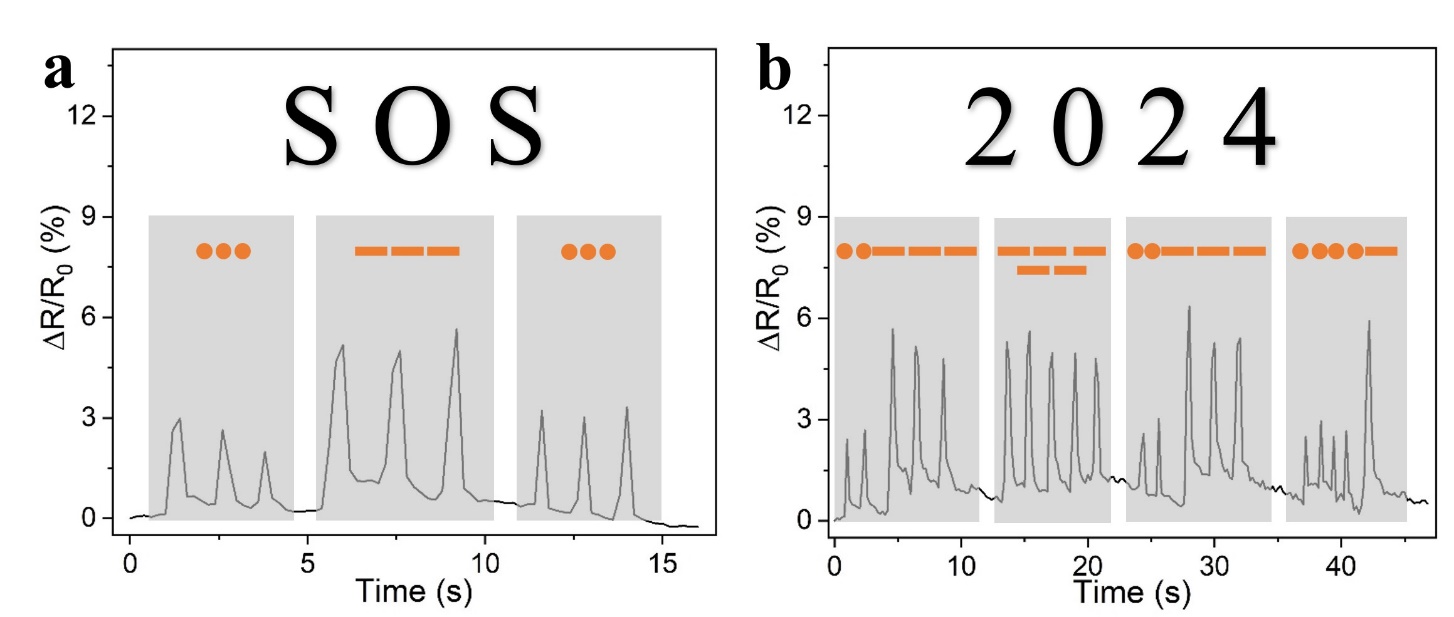


Figure S3. Morse codes of a) ‘SOS’ and b) ‘2024’.

**S5. Mouth breathing monitoring**


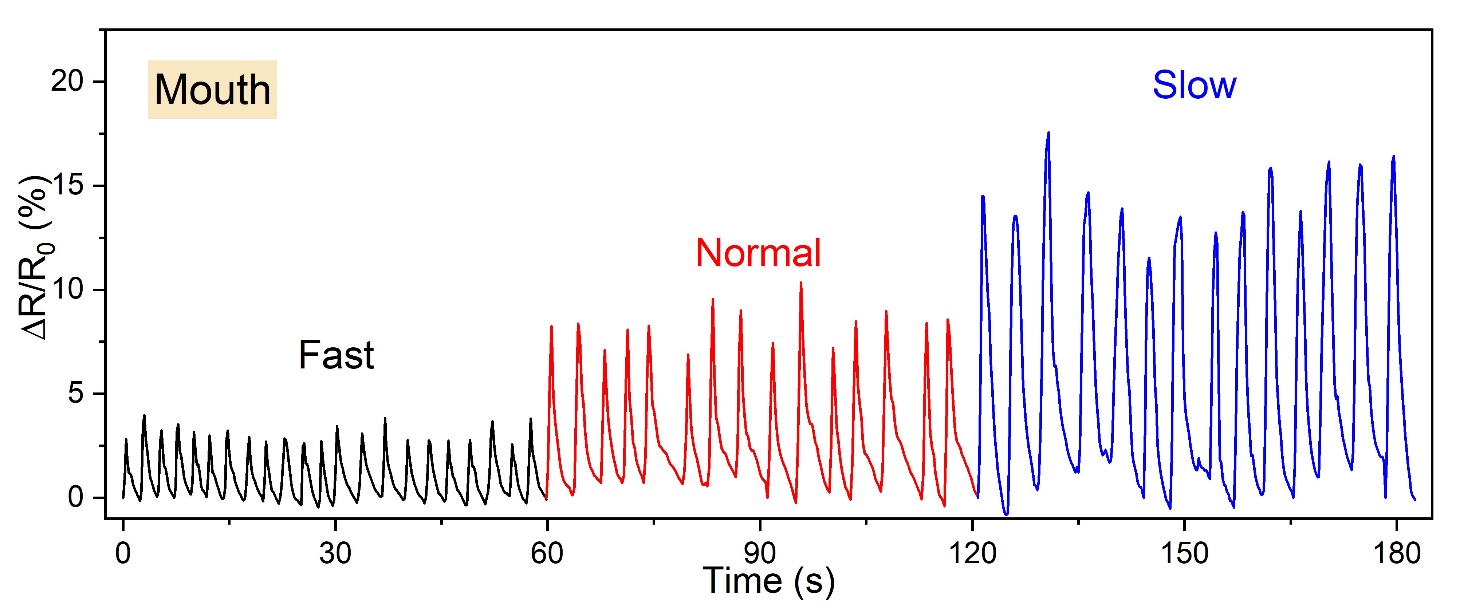


Figure S4. Response curve of the film to mouth breathing under different respiratory rates.

**S6. Edibility of gelatin/charcoal ingredients**

Table S1. Edible contents of gelatin/charcoal ingredients.

| Ingredients | E number | Amount per sensor (mg) | Edibility per day |
| --- | --- | --- | --- |
| Gelatin | E 441 | 36 | >Ten grams^[1]^ |
| Charcoal | E 153 | 4 | Hundreds of milligrams^[2]^ |

**References:**

[1] European Commission - Scientific Committee on Food. "Opinion of the Scientific Committee on Food on specifications for gelatine in terms of consumer health". 27 February 2002. URL: https://ec.europa.eu/food/fs/sc/scf/out122_en.pdf

[2] EFSA Panel on Food Additives and Nutrient Sources added to Food (ANS). Scientific Opinion on the re-evaluation of vegetable carbon (E 153) as a food additive *EFSA Journal* **2012**, 10, 2592.
